# Supplementary material for: Time-dependent enhancement of mRNA vaccines by 4–1BB costimulation
Source: bioRxiv. 2024 Mar 4:2024.03.01.582992. Preprint. [Version 1] doi: 10.1101/2024.03.01.582992 (PMC10942304; doi:10.1101/2024.03.01.582992)
Supplement: 1 [file NIHPP2024.03.01.582992V1-supplement-1.pdf]

bioRxiv preprint doi: https://doi.org/10.1101/2024.03.01.582992; this version posted March 4, 2024. The copyright holder for this preprint (which was not certified by peer review) is the author/funder, who has granted bioRxiv a license to display the preprint in perpetuity. It is made available under aCC-BY 4.0 International license.

Figure S1

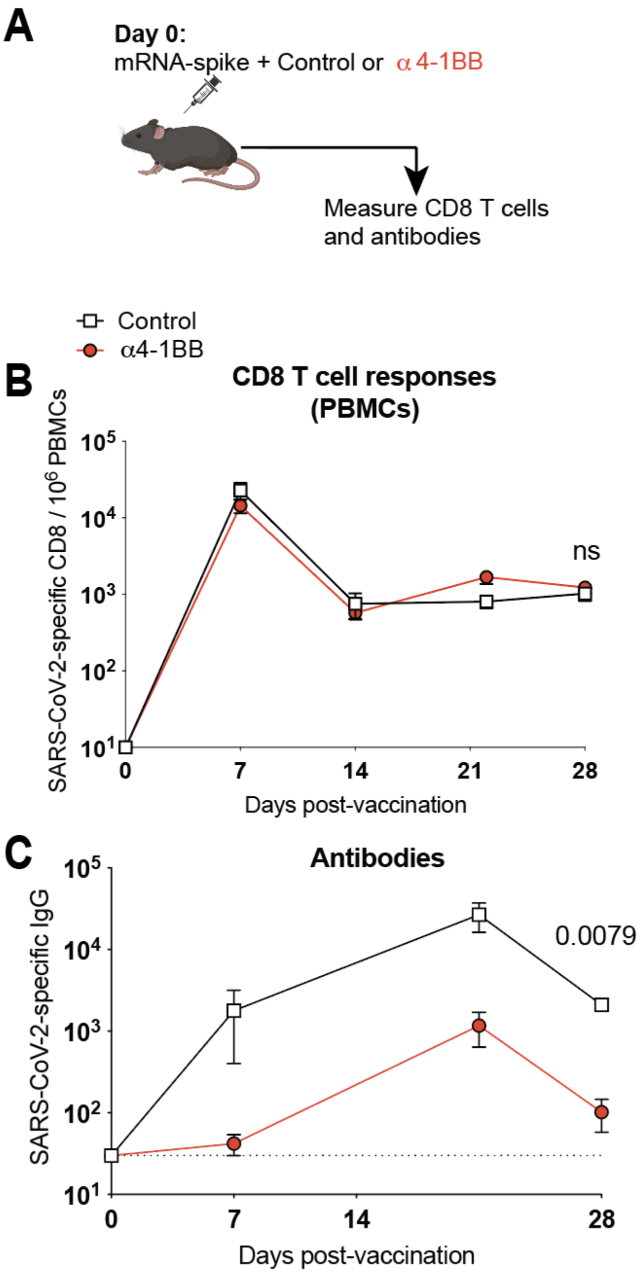

Supp. Fig. 1. 4-1BB costimulation during the acute priming phase does not result in improvement of immune responses following mRNA-SARS-CoV-2 vaccination. (A) Experimental outline for evaluating whether treatment with α4-1BB improves immune responses elicited by an mRNA-SARS-CoV-2 vaccine in C57BL/6 mice. Mice were immunized with 3 μg of a SARS-CoV-2 mRNA spike vaccine followed by α4-1BB or control antibodies at day 0 (on the same of vaccination). **(B)** Summary of SARS-CoV-2-specific CD8 T cell responses in PBMCs. (**C**) Summary of SARS-CoV-2-specific antibody responses in sera. Data are from one experiment, n=5 per group. Indicated *P* values were determined by parametric test (unpaired t test).

bioRxiv preprint doi: https://doi.org/10.1101/2024.03.01.582992; this version posted March 4, 2024. The copyright holder for this preprint (which was not certified by peer review) is the author/funder, who has granted bioRxiv a license to display the preprint in perpetuity. It is made available under aCC-BY 4.0 International license.

Figure S2

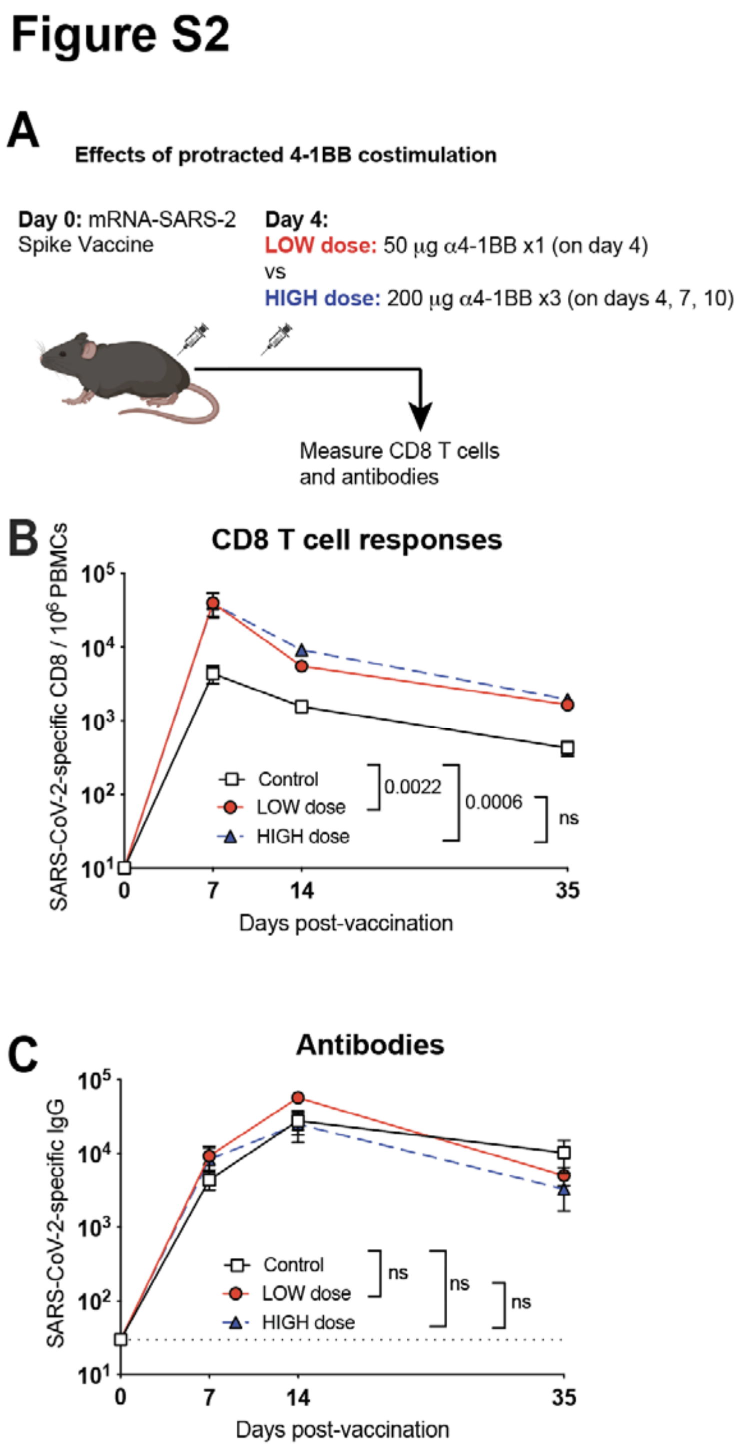

Supp Fig. 2. Long-term 4-1BB costimulation does not result in superior immune responses relative to short-term 4-1BB costimulation. (A) Experimental outline for evaluating the effect of a high dose α4-1BB treatment. Mice were vaccinated with 3 μg of an mRNA-SARS-CoV-2 vaccine. At day 4, one group of mice received a single dose of 50 μg of α4-1BB (low dose); and another group of mice received 200 μg of α4-1BB multiple times (high dose). (**B**) Summary of SARS-CoV-2-specific CD8 T cell responses in PBMCs. (**C**) Summary of SARS-CoV-2-specific antibody responses in sera. Data are from one experiment with n=5 per group. Indicated *P* values were determined by 2-way ANOVA (Dunnett's multiple comparisons tests) at the last time point.

bioRxiv preprint doi: https://doi.org/10.1101/2024.03.01.582992; this version posted March 4, 2024. The copyright holder for this preprint (which was not certified by peer review) is the author/funder, who has granted bioRxiv a license to display the preprint in perpetuity. It is made available under aCC-BY 4.0 International license.

Figure S3

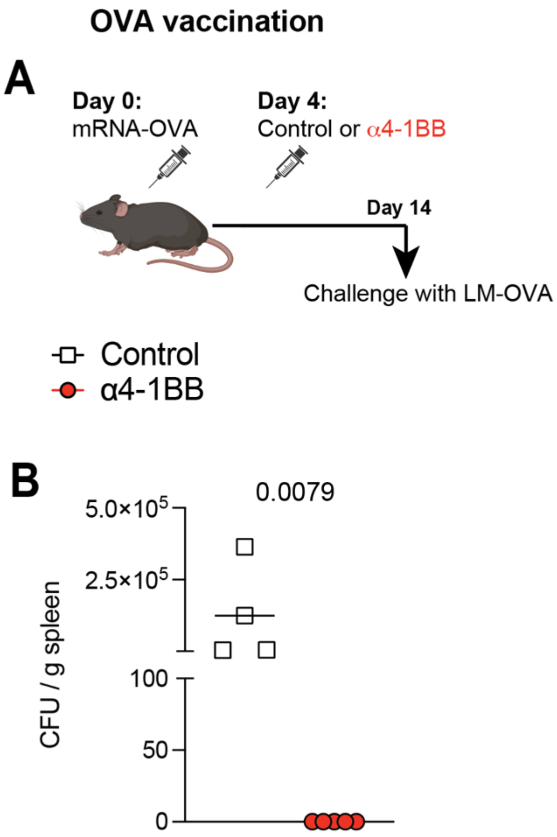

Supp Fig. 3. 4-1BB costimulation following immunization with an mRNA-based listeria vaccine confers sterilizing immunity. (A) Experimental outline for evaluating whether treatment with α4-1BB improves immune responses elicited by an mRNA-OVA vaccine against a listeria-OVA (LM-OVA) challenge. Mice were vaccinated with 3 µg of an mRNA-OVA vaccine followed by treatment with 50 µg of α4-1BB or control antibodies at day 4. At day 14, they were challenged intravenously with a supra-lethal dose of LM-OVA (10^7 CFU) and bacterial loads were quantified. (**B**) Summary of bacterial loads in spleen at day 3 post-challenge. Data are from one experiment, n=4-5 per group. Indicated *P* values were determined by parametric test (unpaired t test).

bioRxiv preprint doi: https://doi.org/10.1101/2024.03.01.582992; this version posted March 4, 2024. The copyright holder for this preprint (which was not certified by peer review) is the author/funder, who has granted bioRxiv a license to display the preprint in perpetuity. It is made available under aCC-BY 4.0 International license.

Figure S4

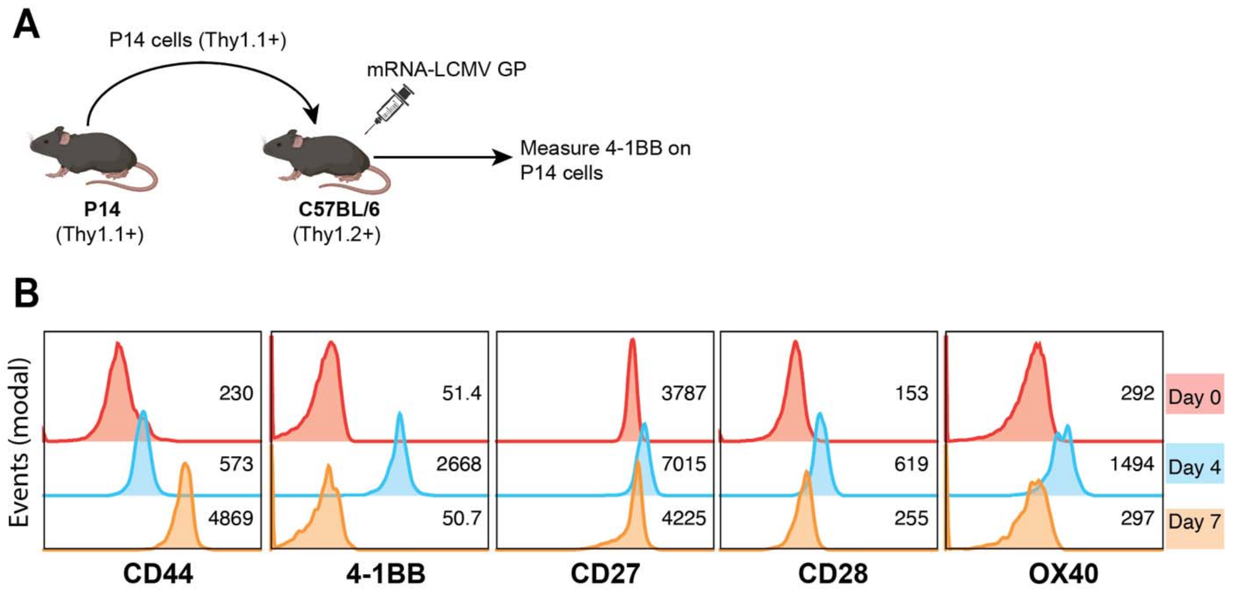

Supp. Fig. 4. 4-1BB is induced on virus-specific CD8 T cells after mRNA vaccination. (A) Experimental outline for evaluating 4-1BB expression following mRNA vaccination. ~35,000 congenically-labeled (Thy1.1+) CD8 T cells from a P14 transgenic mouse were transferred intravenously into a Thy1.2+ C57BL/6 mouse. One day after P14 transfer, C57BL/6 mice were immunized with 3 μg of a mRNA-LCMV GP vaccine, and 4-1BB was measured on virus-specific CD8 T cells at various time points. **(B)** Representative histograms showing 4-1BB and other costimulatory molecule expression on virus-specific (P14) CD8 T cells. CD44 is shown to visualize activationWe utilized this P14 chimera model using high number of P14 cells to allow us to detect 4-1BB expression on virus-specific CD8 T cells at hyperacute points; note that endogenous virus-specific CD8 T cells cannot be detected at hyperacute time points due to their low precursor frequency. Experiment was performed 2 times with n=3 per group, showing similar results (peak of 4-1BB expression at day 4 post-vaccination).

bioRxiv preprint doi: https://doi.org/10.1101/2024.03.01.582992; this version posted March 4, 2024. The copyright holder for this preprint (which was not certified by peer review) is the author/funder, who has granted bioRxiv a license to display the preprint in perpetuity. It is made available under aCC-BY 4.0 International license.

Figure S5

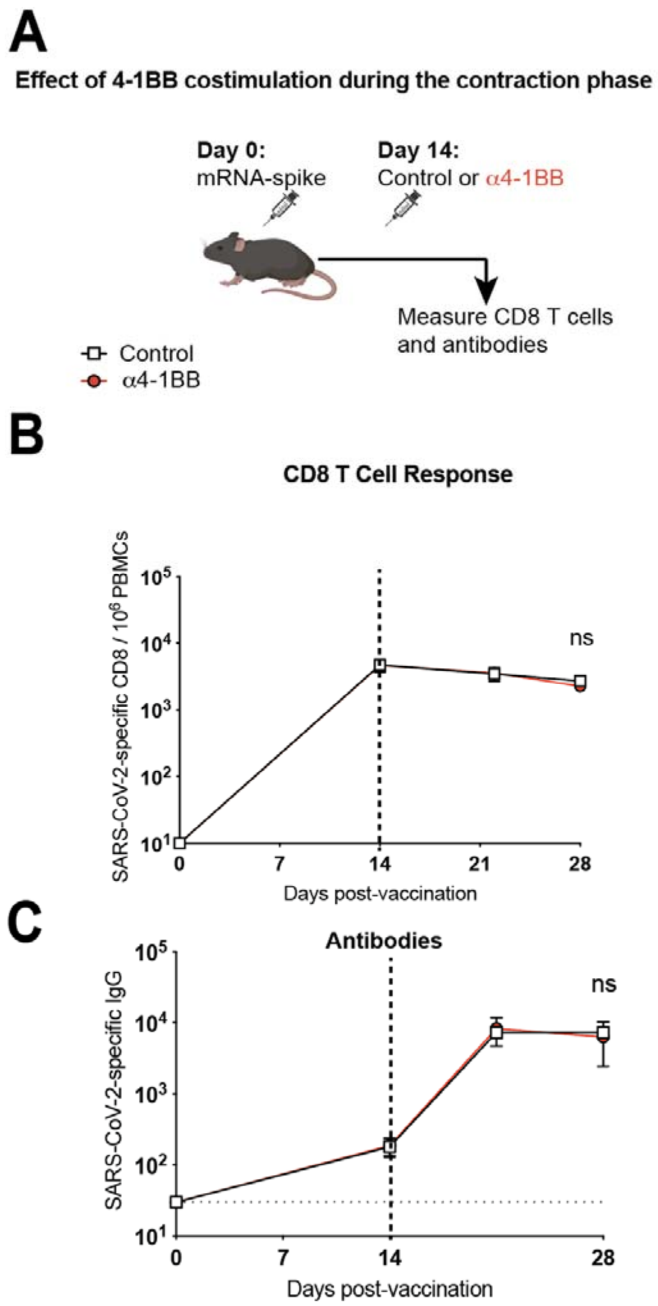

Supp. Fig. 5. 4-1BB costimulation during the contraction phase does not result in improvement of immune responses following mRNA-SARS-CoV-2 vaccination. **(A)** Experimental outline for evaluating whether treatment with α4-1BB improves immune responses elicited by an mRNA-SARS-CoV-2 vaccine in C57BL/6 mice. Mice were immunized with 3 μg of a SARS-CoV-2 mRNA spike vaccine followed by treatment with α4-1BB or control antibodies at day 14. **(B)** Summary of SARS-CoV-2-specific CD8 T cell responses in PBMCs. (**C**) Summary of SARS-CoV-2-specific antibody responses in sera. Data are from one experiment, n=5 per group. Indicated *P* values were determined by parametric test (unpaired t test).
